# Translating Genetic Research into Preventive Intervention: The Baseline Target Moderated Mediator Design

**DOI:** 10.3389/fpsyg.2015.01911

**Published:** 2016-01-07

**Authors:** George W. Howe, Steven R. H. Beach, Gene H. Brody, Peter A. Wyman

**Affiliations:** ^1^Department of Psychology, George Washington UniversityWashington, DC, USA; ^2^Center for Family Research, University of GeorgiaAthens, GA, USA; ^3^Department of Psychiatry, University of Rochester Medical Center RochesterRochester, NY, USA

**Keywords:** prevention research, genes, gene-environment interaction, research design, moderation, mediation

## Abstract

In this paper we present and discuss a novel research approach, the baseline target moderated mediation (BTMM) design, that holds substantial promise for advancing our understanding of how genetic research can inform prevention research. We first discuss how genetically informed research on developmental psychopathology can be used to identify potential intervention targets. We then describe the BTMM design, which employs moderated mediation within a longitudinal study to test whether baseline levels of intervention targets moderate the impact of the intervention on change in that target, and whether change in those targets mediates causal impact of preventive or treatment interventions on distal health outcomes. We next discuss how genetically informed BTMM designs can be applied to both microtrials and full-scale prevention trials. We use simulated data to illustrate a BTMM, and end with a discussion of some of the advantages and limitations of this approach.

## Introduction

**Figure 1:**
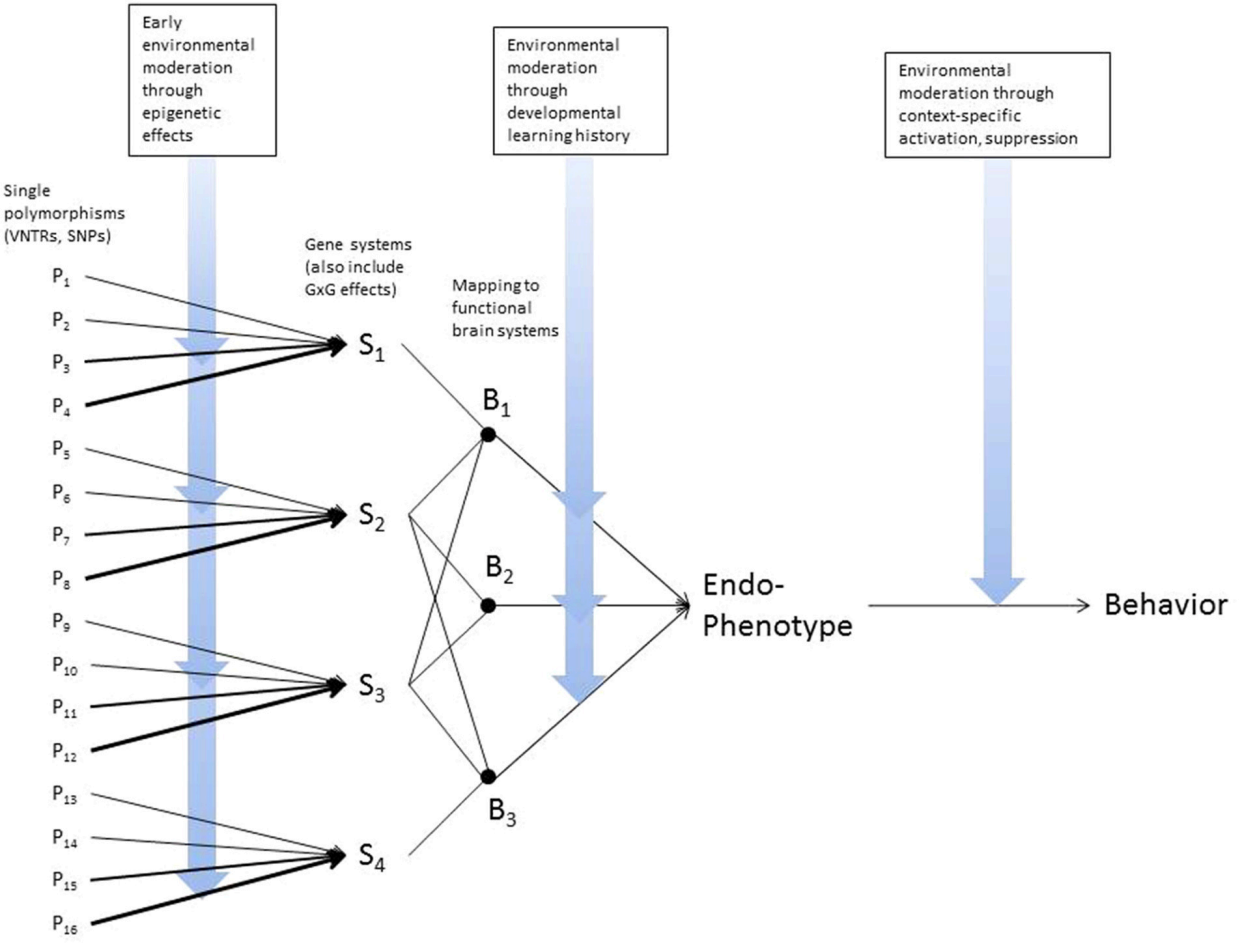
**Dynamic interplay of genes and environments across various levels of analysis**.

As advances accumulate in molecular and behavioral genetics, there is growing interest in translating those findings into practical application. Recent findings from prevention studies bolster this idea. For example, candidate gene variability moderates the impact of family-based interventions designed to reduce risk for adolescent substance use (Brody et al., 2013b) or for early childhood aggression (Bakermans-Kranenburg et al., 2008). This has led to suggestions that genetic moderation may help identify subgroups more likely to benefit from preventive intervention, or alternatively identifying those who will not benefit from preventive intervention. However, we view this outcome as highly unlikely, due to the complex picture of gene-environment dynamics that has emerged over the past decade (Brody et al., 2013a). As Figure 1 illustrates, genetic variation interacts with environmental conditions at several points in the pathway from polymorphic variation to phenotypic behavior, and these pathways also evolve over the course of development. Because preventive interventions target only a subset of these pathways and genetic variation typically moderates the effects of only some specific aspects of preventive intervention, there are always likely to be untargeted pathways and/or pathways not genetically moderated that also contribute to future risk. From this perspective genetically informed research is best viewed as a way to elaborate pathways of risk, provide a window on risk mechanisms, and identify pathways not currently targeted by preventive interventions that may be promising targets for the next generation of prevention trials. Against this backdrop, we suggest that there is a need for a flexible framework to guide translation, taking us from genetic moderation of preventive intervention effects to prescriptive implications for prevention.

In this paper we present and discuss a novel research approach, the baseline target moderated mediation (BTMM) design, that holds substantial promise for advancing this agenda. In particular, we suggest that this design will be more likely to help us answer the general question of which preventive interventions work best for what groups, and why that might be so. We begin by discussing how genetically informed research on developmental psychopathology can be used to identify potential intervention targets. We then describe the BTMM design and its promise in determining whether those proximal targets mediate causal impact of preventive interventions on distal health outcomes, through combining tests of both moderation and mediation in longitudinal designs. We next discuss how genetically informed BTMM designs can be applied to both microtrials and full-scale prevention trials. We end with a discussion of some of the advantages and limitations of this approach.

## Malleable risk and protective mechanisms as proximal targets

Prevention science has a long history of using findings from developmental psychopathology to identify proximal intervention targets. Prevention scientists focus on evidence concerning mechanisms that increase risk for future emotional or behavioral disorders, as well as mechanisms that protect people in the face of such risk. Risk and protective mechanisms must also be placed in developmental context, given evidence that risk trajectories can start early and be modulated by events occurring through childhood and adolescence. And for such mechanisms to be of use for prevention scientists, they must be malleable enough to be altered by intervention technologies (Coie et al., 1993). For example, the Strong African American Families program identified a set of protective mechanisms that could be influenced by working with parents on active parenting of adolescents, and working with youth to help them develop a future orientation and to formulate self-care strategies (Brody et al., 2015). These activities were designed to change cognitions involving intentions to use drugs and positive images of drug-using peers, as well as enhancing self-regulation skills, based on developmental research linking these mechanisms to escalation of drug use.

A second example, the Sources of Strength program, identified peer leader modeling of positive coping behavior as a protective mechanism that can spread through adolescent social networks (Wyman et al., 2010). This program trained peer leaders to provide positive-oriented communications to high school classrooms, sharing narratives about their own use of healthy coping resources as a proximal target for reducing future risk of suicidal behavior in those peer communities (Petrova et al., 2015).

## Genetically informed proximal targets

How can genetic research inform our understanding of malleable risk or protective mechanisms? Within a counterfactual framework (Rubin, 1974), genes are not causes. Or to be more precise, polymorphic variation in candidate genes across a population involves a stable, unchanging background factor (at least until genetic manipulation through gene therapy is possible). Although such factors cannot be considered causes, they can act as causal modifiers. Variation in candidate genes can therefore index variation in the causal structure underlying risk and protection (also referred to as causal effect heterogeneity: Brand and Thomas, 2013), where that structure involves one or more factors that can be manipulated. These malleable factors are of paramount importance for prevention science, as they reflect key targets for interventions designed to prevent future distress or disorder.

We suggest that genetically informed research can be useful for prevention science, to the degree that it tells us something about variation in the impact of risk or protective mechanisms. This can happen in several ways. Experimental studies can test not only whether a specific manipulation can alter a mechanism, but also whether those effects differ depending on genotype. Fox et al. (2011) used a gene-manipulation interaction design to study negative attention bias, a risk factor for anxiety disorder. They were able to modify attention bias in participants having less efficient alleles of the serotonin transporter gene, but not in those with the more efficient allele. Given that participants with both types of alleles are equally at risk for anxiety disorders (Blaya et al., 2007; Gressier et al., 2013; Mak et al., 2015), this suggests that negative attention bias may not be a relevant risk factor for the group carrying the more highly efficient allele. Fox et al. (2011) suggest that the latter group is less prone to negative attention bias in general because they are less influenced by negative experiences over the course of development. This implies that genetic moderation of change in attention bias was mediated by baseline levels of attention bias (emerging through earlier development), with those low in initial attention bias demonstrating less change in subsequent bias than those with initially higher bias [unfortunately Fox et al. (2011) did not test for this effect]. As we discuss later, moderation of effects by baseline target levels may be common in prevention trials, given that change in a target is less likely when an individual or family already has high levels of a protective target or low levels of a risk target.

Fox and other investigators (e.g., Lonsdorf et al., 2009) have used this design to target behavioral or cognitive risk mechanisms, but other genetically informed studies have focused on environmental risk mechanisms. These include observational studies of gene-environment correlation and gene-environment interaction. Several studies have found that specific parent (Klahr et al., 2015) and child (Kryski et al., 2014) genes are associated with parental warmth and hostility, two factors often targeted in interventions to prevent future substance use or other problem behaviors. A rapidly expanding set of studies also document that the association between parenting behavior and child outcomes is moderated by specific child genes including polymorphisms in BDNF (Chen et al., 2015), DRD4 (Cho and Kogan, 2015), and COMT (Sulik et al., 2015). Both sets of findings suggest that preventive interventions that change parenting behavior may have differential effects on this proximal target depending on baseline levels of parenting, which can mediate earlier gene-environment interplay for both parent and child.

Finally, a growing number of studies have included genetic assessments in randomized prevention trials, allowing for tests of whether specific genes moderate the impact of interventions on both proximal targets (Brody et al., 2015) and distal outcomes (Brody et al., 2013b). In most cases, the intervention is found to have less or even no impact on genetically defined subgroups. We suggest that these will often reflect situations where baseline levels of targets involve those with lower risk (or higher protective potential).

In summary, the accelerating research effort incorporating several types of genetically informed designs can point to important potential moderators of intervention impact, guiding us in identifying what works for which people. However, we suggest that this will often be most useful when it identifies malleable proximal targets and tests whether baseline levels of those proximal targets act to moderate intervention effects on both change in proximal targets and change in distal outcomes.

## Baseline proximal targets as moderators

There is growing interest in studying potential moderators of preventive intervention, as a means of learning which interventions work with which people, and under what conditions. Investigators often start with the “usual suspects,” focusing on broad demographic characteristics such as gender, economic condition, or ethnicity. Rather than focusing on such broad factors, we suggest that theoretically “active” moderators, i.e., baseline levels of targeted mediators, will often be more promising to pursue. Consideration of malleable targets that mediate impact on outcomes suggests the strong possibility that baseline levels of the targeted mediator will moderate the impact of the intervention. More broadly, because preventive intervention research is guided by etiologic theories that identify which developmental mechanisms to target and by action theories that identify mechanisms that change those specific targets (MacKinnon et al., 2002), assuming equivalent responsiveness across participants, the impact of the intervention should vary across participants depending on how much room there is for change from the baseline level of the targeted mechanism. Those individuals or families who have higher levels of some targeted risk factor or lower levels of some protective factor have more room to improve, and so, all other things being equal, have greater room for potential improvement in targeted mediators and so greater potential for impact of the intervention on distal health outcomes. Those who begin at lower risk or have more of the protective resource have less room to improve. In more formal terms, baseline levels of an active target should moderate the impact of the intervention on subsequent change in that target.

There are already examples illustrating the basic effect predicted by the BTMM. In the Familias Unidas program family-based interventions were specifically designed to increase positive communication between parents and adolescents in Hispanic families. Accordingly, positive communication was the targeted mediator. In a study that combined data from three randomized trials of the Familias Unidas program, Perrino et al. (2014) found that baseline communication moderated the impact of the intervention, such that those families in the intervention group with poorer communication showed greater increases in positive communication compared to those who began with better communication, while the control group showed no changes in communication regardless of baseline level. Another prevention program working with Mexican American families, the Bridges/Puentes school-based program demonstrated similar effects for several targeted parent behaviors including harshness, positive reinforcement, and monitoring, as well as adolescent active coping and school involvement (Gonzales et al., 2012). These findings were also strengthened through indexing change in the target from baseline to post-test, in these cases using autoregressive modeling.

## Expanding tests of proximal targets as mediators

If the etiologic theory guiding selection of proximal targets is correct, than change in targets should in turn lead to change in more distal outcomes involving behavioral or emotional health. Testing mediational pathways will therefore provide further evidence concerning the validity of both the action and the etiologic theories. This requires that we develop intervention trial designs that allow us to detect changes in both proximal targets and distal outcomes, and that we use statistical techniques that allow for rigorous tests of these theories. Such tests require longitudinal designs that track variation in both target and outcome over developmentally appropriate time periods. Designs employing at least three measurement occasions (baseline, post-test, and follow-up) allow for direct modeling of change in both targets and health outcomes, where change in the former (between baseline and post-test) precedes change in the latter (between post-test and follow-up). Perrino et al. (2014) found that changes in family communication were influenced by the intervention, and in turn post-test communication mediated the impact of the intervention on changes in adolescent internalizing, with better communication associated with decreasing slopes in internalizing as indexed by growth curve models. Gonzales et al. (2012) found evidence of target mediation for several outcomes including adolescent substance use, externalizing, internalizing, and school performance, although not all targets acted as mediators.

An expanded mediational design allows for statistical tests of lagged change-to-change mediation (where change in the proximal target leads to subsequent change in the distal outcome). This model increases plausibility that targeted mechanisms are having a causal impact on outcomes. Modeling the association between change in target and change in outcome is more consistent with a counterfactual account of cause (Morgan and Winship, 2007), particularly when the change in target precedes the change in outcome. Such models allow for testing causal precedence through cross-lagged regression. This method also tests whether earlier changes in the distal outcome may precede changes in the target. If this is not found we can rule out alternate hypotheses that change in outcomes precedes change in targets.

Such models may be of particular use when risk for behavioral or emotional disorders are low at the time of intervention but increase over later stages of development. Most studies we have encountered do not employ true lagged change-to-change models, but rather test whether the target measured at post-test is associated with autoregressive change in the outcome. True lagged change-to-change mediation requires some way of indexing change in target as well as outcome, and can be achieved through the use of latent change models (McArdle, 2009). These models are able to estimate change across two measurement occasions, and therefore require as few as three waves of data to test a change-to-change hypothesis.

## Combining moderation and mediation in the BTMM

**Figure 2:**
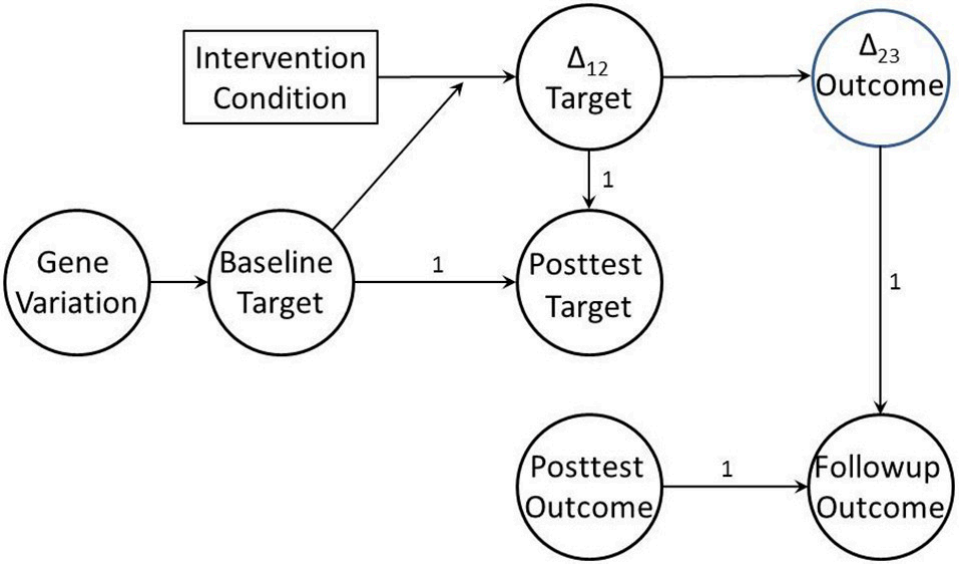
**Baseline target moderated mediation model with lagged change-to-change mediation**. Baseline target, posttest target, posttest outcome, and follow-up outcome are all measured as latent variables. Δ_12_ is a latent variable referencing change in target variable from pretest to posttest; Δ_23_ is a latent variable referencing change in outcome from posttest to follow-up.

If the mechanism we target in a preventive intervention is a true risk or protective mechanism for the distal health outcome, then not only will it mediate the impact of the intervention on that outcome, but that mediation effect itself will vary by target baseline level. This pattern is defined as moderated mediation (James and Brett, 1984). Judd and Kenny (1981) were among the first to apply this concept to the evaluation of intervention trials. As an example, Perrino et al. (2014) demonstrated that the strength of the mediation effect for family communication on adolescent internalizing was highest for those families with the poorest communication at baseline, and decreased as baseline communication quality increased. When combined with change-to-change mediation, moderated mediation provides the basis for the full BTMM design. Figure 2 provides a directed graph for a BTMM model that employs lagged latent change. As we noted earlier, when studying whether a preventive intervention works differently for different subpopulations, most investigators have focused only on general population characteristics such as gender or ethnicity. We suggest that focusing on baseline levels of intervention targets will be more productive in identifying key moderators of intervention effect, and will provide more relevant information for tailoring next generation prevention trials.

## Expanding theoretical precision by identifying multiple proximal targets

Most preventive interventions target more than one risk or protective mechanism in order to maximize impact. When multiple proximal targets are measured, different BTMM models may be necessary depending on whether targets are independent, clustered, or involve chained mediation. Independent targets would have unique and separable impact on health outcomes. In this case etiologic theory would predict that each baseline target would moderate effects only for change in that specific target. The advantage of such a model would be the opportunity to demonstrate specificity by showing both the moderating effects of baseline target levels on change in that target, as well as the lack of moderating effect attributable to baseline levels of other targets. This model has the potential to be powerful and persuasive, but we have been unable to locate any prevention studies employing this approach, probably because etiologic theories do not often identify such independent mechanisms.

Clustered targets involve variables that covary, with proximal targets often influencing each other or jointly indexing the operation of a dynamic system that an intervention is attempting to influence. For example, the Bridges program targeted parent behavior, but also focused on the quality of interactions between parents and adolescents, given that parent harsh behavior covaried with adolescent externalizing (Wong et al., 2014). Mother-adolescent conflict was found to mediate the effect of the intervention on reductions in later adolescent internalizing and externalizing problems (Jensen et al., 2014). This study used a single measure to index the combined effects of parent and adolescent behavior. An alternative approach would be to model the variance shared by a set of proximal targets as a latent variable, and employ the latent variable to estimate both baseline target levels and subsequent change in the targeted mechanism within a BTMM design. For example, Howe et al. (2004), in a study of couples facing unemployment, found that partner reports of support and undermining were strongly correlated. They hypothesized that these variables indexed the quality of dyadic interaction, and used a latent variable model to study the association of the couple-level variable with economic and employment stressors.

Sets of clustered targets may also reflect underlying risk classes that benefit from different components of the preventive intervention. Methods such as latent class analysis can uncover such classes when applied to a set of baseline targets. BTMM analyses using risk classes as the baseline moderator can then be applied to proximal target variables in order to test whether different classes show differential impact due to changes in different sets of proximal targets. Findings of differential effect can then guide development of adaptive interventions that emphasis different proximal targets for different participants.

Chaining (or mediational cascade) involves a series of sequential mediators that transmit the effects of preventive intervention to distal health outcomes. Chaining is common in developmental theories, and is often essential to understanding the effects of early risk or protective mechanisms on later outcomes. This can be of particular importance for health conditions that emerge only at later points in development. For example, McClain et al. (2010) used longitudinal follow-up of a preventive intervention for children of divorced parents to demonstrate that early changes in parent–child relationships were associated with reductions in internalizing, which in turn were associated with reduced risk for externalizing in later adolescence.

## Genetically informed BTMM designs

Baseline target moderated mediation designs can be informed by genetic data in several ways. There has been a recent spate of laboratory experiments demonstrating genetic moderation of highly proximal target response to environmental manipulation. For example Lonsdorf et al. (2009) found that 5-HTTLPR polymorphisms moderate the impact of fear stimuli on fear conditioning but not extinction, while COMT val15met polymorphisms moderated extinction but not conditioning. Growing knowledge of how genes or gene systems are involved in developmental pathways can provide important leads concerning likely targets for preventive intervention.

The malleability and practical utility of such targets can be tested more comprehensively through microtrial methods (Howe et al., 2010) that employ elements of BTMM designs. Microtrials are randomized experiments testing the effects of relatively brief and focused environmental manipulations hypothesized to target and change specific etiologic mechanisms, but not predicted to bring about full prevention effect in distal outcomes. The study of attention bias modification by Fox et al. (2011) used such a design. Although not included in the report, the study could have tested whether baseline values of the target moderated effects on the risk mechanism.

When genes are also measured, this design also allows for tests of whether genetic moderation is in fact due to baseline target moderation, providing an even stronger test of the genetic moderation hypothesis. That is, if genetic variation is correlated with baseline variability in the hypothesized malleable target mechanism, baseline variability in the target mechanism may account for observed genetic moderation effects. Explicating genetic moderation effects in this way would require expanding the BTMM model to test whether variation in baseline target is associated with genetic variation, in effect mediating any gene-by-manipulation interaction on changes in the target. If this association is present, the baseline-by-manipulation interaction is effectively mediating a gene-by-manipulation interaction. Figure 2 illustrates this genetically informed BTMM model.

A key assumption in both laboratory experiments and microtrials is that we have selected a valid target mechanism: that is, these designs do not provide evidence that changing the putative target mechanism will have longer-term impact on distal outcomes, only that the proximal target is malleable, and if it does carry impact, it will do so more strongly for those who need to change in that area. We can however use findings from genetically informed prevention trials to test the distal effects of putative targets, particularly if we incorporate elements of BTMM designs.

As an example, Brody et al. (2015) assessed genetic variation in DRD4 in a randomized trial of the Adults in the Making program, based in part on data suggesting that activation of the dopamine system was associated with selective attention to drug-related cues. They found that DRD4 and a family risk index together moderated the impact of the intervention on drug-related cognitions in youth. Changes in the cognitions were in turn related to changes in subsequent drug use. This model could be further extended through including both baseline levels of cognitions and information on DRD4 variation, and testing whether baseline cognitions mediate the moderating effects of DRD4 on intervention effects on subsequent change in cognition.

Baseline target moderated mediation designs can be used to test whether specific intervention targets are important, so why expand the design to include genetic variation? In particular, to further our understanding of the mechanism itself, findings that baseline target levels do mediate associations with specific genes will suggest that other mechanisms associated with those genes may also be important to explore in order to improve the next generation of prevention trials.

## Example of full BTMM model with simulated data

We used simulation to provide an example of a full BTMM model that incorporates genetic information, and to evaluate estimation bias when the model was estimated with standard structural equation modeling methods. Using the Monte Carlo facility in MPLUS version 7.31 (Muthén and Muthén, 1998–2010), we created 1000 simulated datasets, each with 500 participants. Each dataset simulated data to match that of a clinical trial having roughly equal numbers of participants assigned to either a control or intervention condition (COND). It included scores from three indicators of a putative target (T) measured twice, once at baseline and once at post-test. It also included scores from three indicators of the study outcome (Y) also measured twice, once at post-test and once at follow-up.

For the measurement part of the model, the population model used in the simulation specified two latent variables indexing the target at the two time points, with the three specific indicators loading on their respective latent variables with equal loadings. It also specified similar latent variables indexing outcome at the two time points. Loadings for each latent variable were fixed at one for one indicator, and allowed to vary across datasets for the other two indicators. These latent variables were then used to specify two higher-order latent change variables, one for change in the target and one for change in the outcome, using a standard latent change score model (McArdle, 2009), as illustrated in Figure 2. Cross-time indicators were set to covary with a value of 0.20 for one pair of indicators for each of the two latent change variables, to reflect cross-time conditional dependence often found in such models.

The population model also specified a binary intervention condition variable, with proportion of intervention subjects allowed to vary across trial. Across the 1000 replications the intervention group averaged 44.6% of the total sample. We also constructed a variable to carry information about the interaction of condition with the baseline target latent variable. MPLUS uses a random effects model to allow for such interactions. We also specified a binary variable to simulate information about genetic variability in a candidate gene, such that 15% of the sample carried at least one allele associated with higher levels of the baseline mediator.

The population model specified three regressions in the structural portion of the model. Change in the outcome was regressed on post-test level of the outcome, change in the target, and condition. All three effects were set to a value of 0.25 in the population model, but allowed to vary across the different datasets. Change in the target was regressed on baseline level of the target (parameter = −0.05), condition (parameter = −0.30), and the random effect carrying information on condition by target interaction (parameter = −0.30). These values were chosen to reflect the situation where control and intervention conditions showed little change in the target when baseline values were low, the control group showed almost no increase in rates of change in the target regardless of baseline levels, but the intervention group showed increasing rates of change as baseline levels increased. These effects reflect the pattern of moderated mediation predicted when an intervention successfully shapes a putative target. And finally, the baseline target was regressed on the genetic variable, with a parameter of 0.30.

We used the MPLUS Monte Carlo facility to estimate and combine results from all 1000 datasets, using the model illustrated in Figure 2, and including correlations among cross-time indicators as well as the regression of baseline target on genetic variation (which do not appear in the Figure). We used the standard robust maximum likelihood estimator with numerical integration. Aggregated results provide information about potential bias and coverage of the model. Bias in the measurement model parameters (0.23% or below) and associated standard errors (2.63% or below) are very low and coverage (0.93–0.96) is excellent. Estimates for the structural model are shown in Table 1. Bias is also low; with one exception, bias scores for parameters are below 2.2% and for standard errors below 3.4%. The regression parameter indexing the association between the baseline mediator and change in mediator shows higher bias (5.4%), likely due to the small effect size of this parameter. Coverage is again excellent, ranging from 0.94 to 0.96. These findings indicate that the BTMM model can be specified and estimated with accuracy using standard structural equation modeling approaches.

**Table 1:** **Estimates of bias and coverage for selected parameters from Monte Carlo simulation of data from a genetically informed baseline target moderated mediation study**.

			**Model parameters**			**Model standard errors**			
			**Population**	**Estimate**	**Bias (%)**	**Population**	**Estimate**	**Bias (%)**	**Coverage**
**Measurement model**									
Target	Tl	BY							
(pretest)	T1A		1	1	0.00	0	0		
	TIB		1	1.001	−0.10	0.0533	0.0522	2.06	0.93
	TIC		1	1.0022	−0.22	0.0525	0.0516	1.71	0.95
Target	T2	BY							
(post-test)	T2A		1	1	0.00	0	0		
	T2B		1	1.0002	−0.02	0.0387	0.0381	1.55	0.95
	T2C		1	1.0007	−0.07	0.0372	0.0378	−1.61	0.96
Outcome	Y2	BY							
(post-test)	Y2A		1	1	0.00	0	0		
	Y2B		1	1.0022	−0.22	0.0532	0.0518	2.63	0.94
	Y2C		1	1.0023	−0.23	0.0504	0.0514	−1.98	0.96
Outcome	Y3	BY							
(Followup)	Y3A		1	1	0.00	0	0		
	Y3B		1	1.0001	−0.01	0.0297	0.0295	0.67	0.95
	Y3C		1	1.0009	−0.09	0.0299	0.0293	2.01	0.94
**Structural model**									
Change in outcome	DEL23Y	ON							
	Y2		0.25	0.2549	−1.96	0.0707	0.0731	−3.39	0.95
	DEL12T		0.25	0.2502	−0.08	0.058	0.057	1.72	0.95
	COND		−0.25	−0.2517	−0.68	0.1046	0.1059	−1.24	0.96
Change in target	DEL12T	ON							
	Tl		−0.05	−0.0473	5.40	0.0863	0.0838	2.90	0.95
	CONDXT1		−0.3	−0.2968	1.07	0.1074	0.1056	1.68	0.94
	COND		−0.3	−0.3066	−2.20	0.1104	0.1092	1.09	0.95
Pretest mediator on genetic variation	Tl	ON							
	G		0.3	0.298	0.67	0.1299	0.1319	−1.54	0.96

## Limitations and conclusions

Baseline target moderated mediation designs capitalize on random assignment to prevention condition in order to buttress causal inference concerning intervention effects on both proximal targets and distal health outcomes. However, this does not extend to tests of moderation or mediation (VanderWeele, 2015). Significant moderator effects do provide strong evidence of causal heterogeneity, but conclusions about the sources of that heterogeneity are open to confounding. For example, moderating effects of baseline family communication could be due to some other historical variable that influences baseline family communication and also acts as the true moderator of intervention impact.

Similar issues arise concerning paths from mediators to outcomes (Imai et al., 2010). However, the complex moderated mediator hypothesis, if supported, would increase plausibility of causal inference because it is more difficult to find plausible confounds that fit this pattern. For example, we might posit that an intervention could have an impact on outcomes through changing some other proximal target that influences both our putative target and subsequent outcome, but it seems less plausible that the effects on the second target would be moderated by baseline levels of the first. And as discussed earlier, we can also include a variety of design elements to further bolster plausibility of causal impact, including lagged change-to-change assessment, cross-lagged analysis, and inclusion of multiple targets to test target-specific moderated mediation.

A number of years ago Sandler et al. (1991) advocated using research on risk and protective mechanisms to identify subpopulations at risk for future emotional and behavioral problems, and developing interventions that specifically targeted those mechanisms in those groups. We see the BTMM design as a useful tool for advancing and refining this aim. For example, if we find that some families are already good at communicating with adolescents (Perrino et al., 2014), and that those families do not gain long-term benefit from a prevention program through proximal changes in their communication, we can revise future programming for that particular subgroup to focus more intensively on targets that are relevant for them, based on tests of other proximal targets.

In summary, we suggest that BTMM designs and associated statistical models hold great promise for translating studies of gene-environment dynamics into prevention science. They also provide a means of testing how and when specific proximal targets of preventive intervention will have maximal impact on distal health outcome, and as a result can guide refinement of next generation prevention trials. And, given current standards for measuring both targets and outcomes at baseline as well as at post-test and follow-up, they can be easily implemented within current prevention trial designs with little or no extra cost.

### Conflict of interest statement

The authors declare that the research was conducted in the absence of any commercial or financial relationships that could be construed as a potential conflict of interest.
